# SMIFormer: Learning Spatial Feature Representation for 3D Object Detection from 4D Imaging Radar via Multi-View Interactive Transformers

**DOI:** 10.3390/s23239429

**Published:** 2023-11-27

**Authors:** Weigang Shi, Ziming Zhu, Kezhi Zhang, Huanlei Chen, Zhuoping Yu, Yu Zhu

**Affiliations:** 1 School of Automotive Studies, Tongji University, Shanghai 201804, China; 1811128@tongji.edu.cn (W.S.); yuzhuoping@tongji.edu.cn (Z.Y.); 2 School of Information Science and Engineering, East China University of Science and Technology, Shanghai 200237, China; zimingzhu@mail.ecust.edu.cn (Z.Z.); y30220946@mail.ecust.edu.cn (K.Z.); 3 Shanghai Motor Vehicle Inspection Certification & Tech Innovation Center Co., Ltd., Shanghai 201805, China; huanleic@smvic.com.cn

**Keywords:** 4D imaging radar, point cloud, 3D object detection, voxel feature decoupling, multi-view feature interaction, deep learning, autonomous driving

## Abstract

4D millimeter wave (mmWave) imaging radar is a new type of vehicle sensor technology that is critical to autonomous driving systems due to its lower cost and robustness in complex weather. However, the sparseness and noise of point clouds are still the main problems restricting the practical application of 4D imaging radar. In this paper, we introduce SMIFormer, a multi-view feature fusion network framework based on 4D radar single-modal input. SMIFormer decouples the 3D point cloud scene into 3 independent but interrelated perspectives, including bird’s-eye view (BEV), front view (FV), and side view (SV), thereby better modeling the entire 3D scene and overcoming the shortcomings of insufficient feature representation capabilities under single-view built from extremely sparse point clouds. For multi-view features, we proposed multi-view feature interaction (MVI) to exploit the inner relationship between different views by integrating features from intra-view interaction and cross-view interaction. We evaluated the proposed SMIFormer on the View-of-Delft (VoD) dataset. The mAP of our method reached 48.77 and 71.13 in the fully annotated area and the driving corridor area, respectively. This shows that 4D radar has great development potential in the field of 3D object detection.

## 1. Introduction

The conventional automotive radar has been widely used in advanced driver assistance systems (ADAS) and autonomous driving [1], but it faces limitations such as the absence of elevation information and low resolution compared with LiDAR-based perception technologies [2,3,4,5,6]. To overcome these limitations, the development of 4D imaging radar [7] has emerged as a promising solution. Unlike conventional radar, 4D imaging radar can measure the pitch angle, which allows for the acquisition of elevation information. This additional dimension enhances the understanding of the environment and improves the accuracy of object detection and localization. The literature [8,9] extensively explores the evolution and performance of 4D radar technology, with various studies highlighting advancements and discussing different brands in this field. Short-range sensing applications specifically demonstrate the exceptional performance of 4D radar [10].

When compared with LiDAR, perception systems based on 4D imaging radar offer several advantages. These systems have lower hardware and maintenance costs for object detection, making them more cost-effective. Moreover, they exhibit resilience to external factors. LiDAR point cloud and 4D radar point cloud share similarities, but they also have notable differences; 4D imaging radar enables velocity measurements, enhancing the original point cloud data by providing additional dimensions to represent the vector velocity information of each point. While both technologies provide valuable data for perception systems, LiDAR’s inability to penetrate fog and dust hinders its application in severe weather [11], and 4D radar measurements are subject to noise due to multi-path propagation and limited angular resolution caused by longer wavelengths and a restricted number of antennas. This limited number of antennas in 4D radar results in a sparser point cloud compared with the denser point cloud produced by LiDAR systems [12]. Consequently, 4D radar captures less geometry and semantic information compared with LiDAR. This difference in point cloud density poses a challenge for existing 3D object detection algorithms, which are specifically designed for dense LiDAR point clouds. As a result, these algorithms may not perform optimally when directly applied to sparse 4D radar point cloud data.

**Figure 1:**
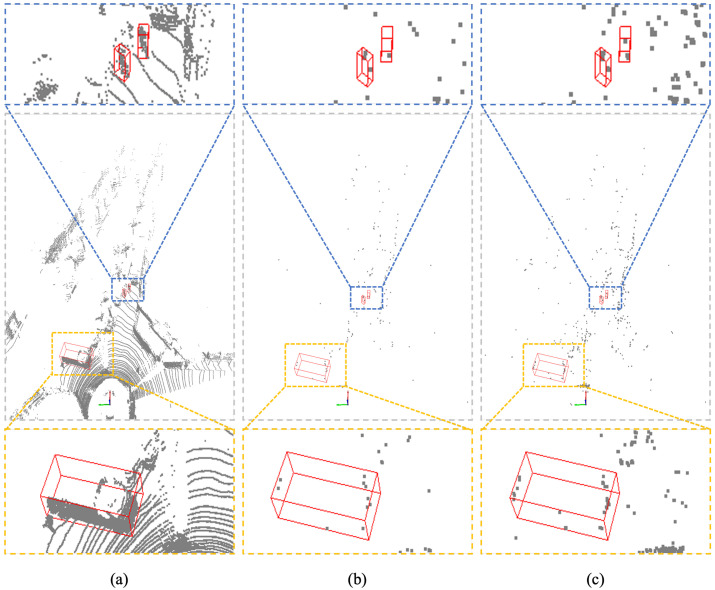
Point cloud visualization collected by different sensors. (**a**) LiDAR. (**b**) 4D Radar single frame. (**c**) 4D Radar 5 frames.

Currently, methods based on LiDAR point clouds often employ voxel-based representations, where the 3D space is divided into voxels and each voxel is assigned a vector to indicate its status. However, the sheer number of voxels presents computational challenges, necessitating specialized techniques such as sparse convolution [13]. In outdoor scenes, the distribution of information is not uniform. To address this, modern methods based on LiDAR point clouds collapse the height dimension and focus on the bird’s-eye view (BEV), as this perspective exhibits the most variability in information [14,15,16,17,18,19,20]. These methods encode the 3D information of each object in the BEV grid. BEV-based methods excel in LiDAR 3D object detection tasks due to their ability to predict coarse-level bounding boxes for commonly encountered objects [16,17]. Furthermore, as depicted in Figure 1, the dense nature of the Lidar point cloud allows for a more comprehensive acquisition of an object’s surface profile compared with the 4D radar point cloud. On average, objects in a 4D imaging radar point cloud can only be assigned to less than 200 points, which poses a challenge in encoding the extremely sparse outline information of objects with various 3D structures using a flattened vector.

To develop a safer and more robust 4D radar autonomous driving system, a more comprehensive and fine-grained understanding of the 3D surroundings is necessary. The generalization of BEV to model fine-grained 3D structures while maintaining efficiency and detection performance remains an open question. In other words, the use of spatial geometry information has become a key factor in improving the accuracy and performance of the 3D object detection field of 4D radar. In order to enhance 3D surrounding perception, it is essential to effectively represent 3D scenes.

In this paper, we propose a method that aims to retain and incorporate multi-view geometric information into spatial features for more robust object detection. Recognizing the untapped potential within multi-view geometric data, we seek to bridge this gap by incorporating it into spatial features for object detection purposes. By considering multiple views simultaneously, we aim to capture a more comprehensive understanding of objects from different angles or orientations. This approach holds great promise for improving both accuracy and performance across diverse scenarios. We take inspiration from previous research but introduce novel techniques specifically designed to retain and incorporate multi-view geometric information effectively. By integrating data from multiple viewpoints during feature extraction processes, we create richer representations that enable more precise identification and localization of objects within complex scenes.

By leveraging multi-view geometric information, our method offers several potential benefits towards achieving enhanced accuracy or improved overall performance in object detection tasks: Robustness: Considering multiple views helps mitigate occlusion issues commonly encountered when analyzing 3D scenes. By capturing information from various angles, our method can better handle instances where objects may be partially or fully obscured from certain perspectives. Discriminative Power: The inclusion of multi-view geometric features provides additional discriminative power in distinguishing between objects that may share similar appearances but differ in their spatial characteristics. This added information aids in reducing false positives and improving detection precision. Generalization: Incorporating multi-view perspectives promotes greater generalization capabilities, enabling the model to adapt more effectively to unseen or novel scenarios by leveraging a broader range of spatial representations. The main contributions of our work can be summarized as follows:

- We addressed the issue of insufficient representation of extremely sparse point clouds in a single view by decoupling 3D voxel features into separate front view (FV), side view (SV), and bird’s-eye view (BEV) planes.
- We propose sparse-dimensional compression as an alternative to dense-dimensional compression. By individually placing voxels on each view plane and aggregating features at corresponding positions, we construct a two-dimensional sparse feature matrix. This approach allows us to achieve precise predictions while minimizing memory and computational demands.
- We suggest multi-view feature interaction (MVI) as a method to enhance spatial perception. MVI divides the full-size feature map into non-overlapping windows, enabling more effective interaction between view-inside and view-outside features through self-attention and cross-attention. This approach enhances spatial perception at each interaction level with only a small increase in computation.

## 2. Related Work

### 2.1. 3D Object Detection with LiDAR Point Cloud

Voxel-based and pillar-based feature representations are the main methods of relevant prior grid-based 3D detection work using LiDAR point clouds.

VoxelNet [21] stands as one of the pioneering studies that employ end-to-end 3D detection by dividing a point cloud into voxels and accurately predicting 3D bounding boxes. While it offers promising results in object recognition tasks, its high computational cost limits its applicability for real-time applications. SECOND [22] introduces an innovative approach through the utilization of 3D sparse convolution to enhance performance while reducing computational overheads. By extracting voxel features using a backbone network with sparse convolution techniques, these features are then concatenated along the height dimension before undergoing additional processing via 2D convolution layers to obtain dense bird’s-eye view (BEV) features. This methodology significantly accelerates computation speed without compromising accuracy. Building upon the foundations laid by SECOND [22], CenterPoint [2] proposes a novel idea of employing a single positive cell for each detected object. By refining existing methodologies further, CenterPoint aims to streamline detection pipelines by optimizing computations involved in identifying individual objects within crowded scenes effectively. The method known as VISTA [23] takes another step forward by projecting 3D feature maps onto both bird’s-eye view (BEV) and range view (RV). Through this multi-view transformer fusion approach, VISTA achieves enhanced mapping capabilities for improved object recognition across different perspectives. Recognizing the importance of foreground data during learning processes led researchers to develop FocalsConv, a dynamic mechanism that dynamically focuses on foreground information [24]. By selectively emphasizing relevant data, FocalsConv aims to refine the learning process and enhance detection accuracy. Transformer [25] is a powerful global modeling method. CenterFormer [26] improves the prediction accuracy of bounding boxes by aggregating features around the center candidate and introducing this architecture into the detection head. The majority of existing methods (specifically [2,23,24,27,28]) have been developed based on the framework established by SECOND [22]. While these methods have shown promise in various object detection tasks, they fall short in effectively leveraging multi-view geometric information. By solely relying on single perspectives or views, these approaches overlook potentially significant details that could enhance their accuracy and overall performance.

PointPillars [29] is a pioneering pillar-based approach that utilizes PointNets [30] to encode point features before transforming them into a pseudo-image in bird’s-eye view (BEV) using pooling operations. By employing only 2D convolutional layers, it enables end-to-end learning while making it suitable for computation-constrained embedded systems with low latency requirements. Infofocus [31] enhances PointPillars [29] by introducing a second-stage attention network specifically designed for fine-grained proposal refinement. This additional network improves precision and accuracy by focusing on important details within detected objects. Based on CenterPoint-pillar [2] architecture, PillarNet [32] incorporates the ResNet18 structure with 2D sparse convolution into its backbone for BEV feature extraction. This modification further enhances computational efficiency while maintaining high performance levels. Experiments have demonstrated that after sufficient extraction through 2D sparse convolution techniques, the accuracy achieved by pillar-based networks can be comparable to voxel-based methods commonly used in 3D object detection tasks. However, this accomplishment comes at a cost; pillar-based approaches suffer from significant loss of critical 3D geometric information during transformation processes. Due to the inherent nature of pillarization and subsequent projection onto BEV representations, these approaches struggle to overcome limitations associated with preserving essential aspects related to 3D object detection. The loss of geometric information poses challenges in accurately localizing objects and understanding their spatial relationships within the scene.

### 2.2. 3D Object Detection with 4D Imaging Radar Point Cloud

In the past period of time, despite the fact that the traditional radar was cheaper than lidar and was robust in complex weather, it was unable to provide sufficient support for 3D object detection due to its point cloud being too sparse and lacking elevation information. However, with the maturity of 4D millimeter wave imaging radar technology, the density of radar point clouds has been significantly improved, and it can provide elevation information like lidar and additional speed information. This allows people to see the potential of using 4D radar for 3D object detection.

Based on pillarization inspired by PointPillars [29], RPFA-Net [33] designs a self-attention module for sparse columnar features to better globally model sparse features. RadarMFNet [34] compensates the speed information of the radar point cloud by estimating the speed information of the ego vehicle and matches the multi-frame millimeter wave imaging radar point cloud to the last frame, using a multi-frame radar point cloud for 3D object detection. SMURF [35] Introducing kernel density estimation into radar point clouds, using additional statistical features to effectively mitigate the adverse effects of inherent noise and sparsity in point clouds. Furthermore, research on multi-modal fusion of camera images has also been applied to radar point clouds. RCBEV [36] uses the camera image as the forward view (FV) and the radar point cloud as the BEV for multi-view fusion. CRN [37] uses radar point clouds for space occupancy prediction to better guide the conversion of camera image features into BEV features. RCFusion [38] lifts camera image features to 3D radar point cloud coordinates through orthogonal feature transformation (OFT) to achieve multi-modal fusion. LXL [39] uses sampling-based methods to lift 2D image features to 3D point cloud space and further optimize the lifted features through depth prediction and occupancy prediction.

### 2.3. Point Cloud Perception for Indoor Scenes

The change in feature extraction method brought about the change in point cloud density can also be extended to indoor scenes. Indoor scenes tend to have a higher density of point clouds than outdoor lidar, let alone 4D imaging radar. That is, we can gain insight into how the 3D scene is represented by observing the difference in the way features are extracted as point cloud sparsity decreases.

Poly-PC [40] proposes a Res-SA layer for efficient scaling and a weight-entanglement-based one-shot NAS technique to find optimal architectures, with a task-prioritization-based gradient balance algorithm for training and high performance in various tasks. CO-Net [41] uses a Res-MLP block for feature extraction, a nested layer-wise processing policy for optimal architecture, and sign-based gradient surgery to promote training and optimize task-shared parameters in multi-task learning on point clouds. FARP-Net [42] introduces an LGFAL layer that combines local and global features using an attention-based fusion module, incorporates an LFAM to map local features into a normal distribution, and proposes a WRPM for object proposal generation that weighs relation importance among object candidates for improved proposal quality. Auto-Points [43] uses a SAM layer for flexible scaling, simplifies architecture search with child candidates, and optimizes them using a weight-entwinement NAS technique for each point cloud task.

## 3. Method

**Figure 2:**
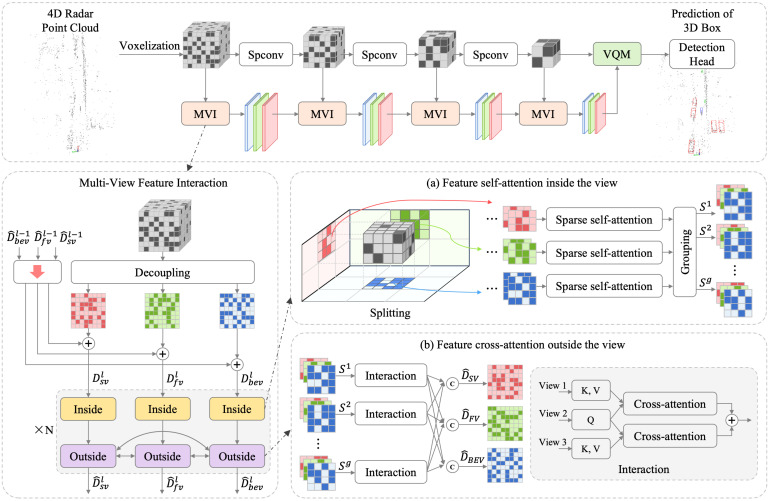
The overall architecture of our proposed SMIFormer. Our approach employs a 4D Imaging Radar point cloud as input for 3D object detection. To enable multi-view feature encoding and interaction, we introduce a parallel fusion model. Decoupled feature maps are colored by view directions with blue for bird’s-eye view, red for side view and green for front view.

In LiDAR-centric autonomous driving perception tasks, it is common to use a 3D convolutional backbone network for extracting features from the point cloud. These features are then inputted into a dedicated encoder based on the presentation framework. To capture more spatial context information in sparser 4D imaging radar point clouds (over 200 times sparser than LiDAR point clouds), we introduce SMIFormer. Figure 2 showcases the structure of SMIFormer, which proposes the utilization of a transformer-based multi-view encoder. This encoder utilizes the attention mechanism to enhance the point cloud feature in the multi-view plane.

### 3.1. Framework Overview

In SMIFormer, we propose the incorporation of decoupled per-view feature encoding, feature self-attention inside the view, and feature cross-attention outside the view. These techniques are employed to efficiently extract multi-view plane features. Each view plane represents a mesh cell feature associated with one of the three planes, capturing specific view information from the corresponding pillar area. The feature self-attention inside the view focuses on encoding in-view features by interacting with view planar features within the same view. On the other hand, feature cross-attention outside the view facilitates direct interaction between view plane features across different views, allowing for the incorporation of richer contextual information from all perspectives.

### 3.2. Decoupled Per-View Feature Encoding

To generate a comprehensive observation of the scene from different perspectives and overcome limitations such as object occlusion caused by solely extracting bird’s-eye view features, we compress the voxel features into separate front view (FV), side view (SV), and bird’s-eye view (BEV) planes when constructing the three-view features.

The 3D object detectors presented in papers [2,22,29] employ compression techniques to convert sparse 3D voxel features into dense 2D maps. This is achieved by transforming sparse features into dense ones and incorporating altitude information (along the z-axis) into the channel dimension. However, these operations require additional memory and computational resources.

In order to achieve precise predictions while minimizing memory and computational demands, we utilize sparse dimension compression to separate the features of each view. This involves placing voxels individually on each view plane and aggregating the features at corresponding positions to construct a two-dimensional sparse feature matrix $\left(D_{bev},D_{fv},D_{sv}\right)$. The construction of this matrix relies on the values of sparse features and their coordinate indexes. After performing sparse dimension compression, the calculation of sparse features $F_{v}$ and their corresponding coordinates $\left(C_{bev},C_{fv},C_{sv}\right)$ is as follows:

(1) $$\begin{array}{cc} C_{bev} & =\left\{\left(x_{c},y_{c},0\right)|c\in C_{voxel}\right\}\\ C_{fv} & =\left\{\left(x_{c},0,z_{c}\right)|c\in C_{voxel}\right\}\\ C_{sv} & =\left\{\left(0,y_{c},z_{c}\right)|c\in C_{voxel}\right\} \end{array}$$

(2) $$\begin{array}{cc} F_{v} & =\left\{\sum_{c\in A_{\hat{c}}}f_{c},|\hat{c}\in C_{v}\right\},v\in \left[bev,fv,sv\right]\\ A_{\hat{c}} & =\left\{c|x_{c}=x_{\hat{c}},y_{c}=y_{\hat{c}},z_{c}=z_{\hat{c}},c\in C_{v}\right\} \end{array}$$

### 3.3. Voxel Feature Query Module

**Figure 3:**
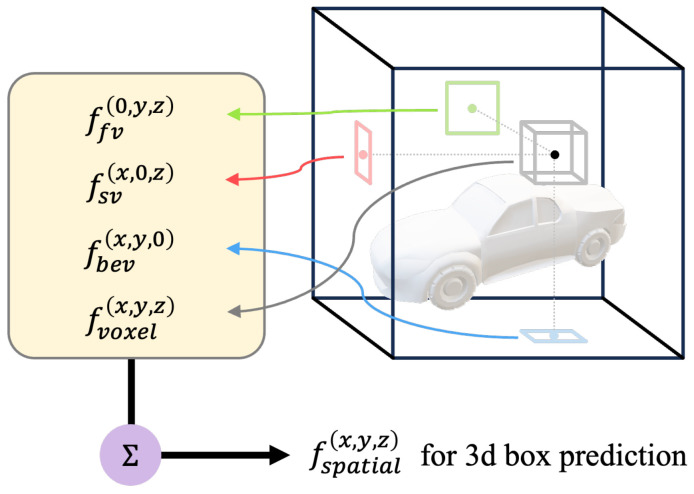
Voxel feature query module. Given a specific voxel location, we project its 3D coordinates onto each of the three axially aligned planes (BEV, SV and FV). Afterwards, we extract the features of each projection from a sparse 2D feature map and consolidate the resulting features into a single feature vector.

The decoupled multi-view representation aims to provide a comprehensive description of a query voxel positioned at (x,y,z) in three-dimensional space. This is accomplished by consolidating its projections on BEV, SV, and FV views, as illustrated in Figure 3.

Specifically, we initiate the process by projecting the voxels onto the BEV, SV, and FV view planes, thereby obtaining the coordinates [(x,y,0),(x,0,z),(0,y,z)]. Subsequently, we sample the BEV, SV, and FV planes at these coordinates to retrieve the corresponding features $\left[f_{bev}^{\left(x,y,0\right)},f_{sv}^{\left(x,0,z\right)},f_{fv}^{\left(0,y,z\right)}\right]$:

(3) $$\begin{array}{c} f_{bev}^{\left(x,y,0\right)}=I\left(F_{bev},T_{bev}\left(x,y,0\right)\right)\\ f_{sv}^{\left(x,0,z\right)}=I\left(F_{sv},T_{sv}\left(x,0,z\right)\right)\\ f_{fv}^{\left(0,y,z\right)}=I\left(F_{fv},T_{fv}\left(0,y,z\right)\right) \end{array}$$

By combining these three features with the voxel’s intrinsic features, we generate the final $f_{spatial}^{\left(x,y,z\right)}$:

(4) $$f_{spatial}^{\left(x,y,z\right)}=SUM\left(f_{voxel}^{\left(x,y,z\right)},f_{bev}^{\left(x,y,0\right)},f_{sv}^{\left(x,0,z\right)},f_{fv}^{\left(0,y,z\right)}\right)$$

The variables $F_{bev}$, $F_{sv}$, and $F_{fv}$ are feature maps represented by tensors with dimensions $R^{W_{bev}\times H_{bev}\times D}$, $R^{W_{fvsv}\times H_{fvsv}\times D}$, and $R^{W_{fvsv}\times H_{fvsv}\times D}$, respectively. The variable *f* is a feature vector represented by a tensor with dimensions $R^{1\times D}$. *I* represent the index function that samples the feature vectors from the feature maps of BEV, SV, and FV based on the given coordinates. The coordinate transformation function, denoted as *T*, is defined as follows: Considering that the BEV, SV, and FV view planes align with the axes of three-dimensional space, each projection function *T* only needs to perform a straightforward index dimensionality reduction on the two associated coordinate systems it encompasses.

### 3.4. Splitting for Multi-View Feature Interaction

The aforementioned decoupled per-view feature encoding structure compresses the sparse three-dimensional feature voxels into three views, utilizing the feature map as the representation of each perspective to enable multi-perspective feature interaction. However, due to the high-resolution nature of each view plane feature (approximately $10^{5}$ queries), it becomes inefficient and feature-redundant to compute full-scale attention directly within the same view, considering the significant computational costs and GPU memory requirements. To address this issue, we propose a windowing strategy to temporally divide the full-size feature map into non-overlapping windows, with each window covering a small area of the feature map. Specifically, the two-dimensional sparse feature map with spatial dimensions W×H is evenly divided into $g=g_{w}\times g_{h}$ groups, where each group has spatial dimensions $W^{\prime }\times H^{\prime }$ (assuming $W=W^{\prime }\times g_{w},H=H^{\prime }\times g_{h}$). This paper’s windowing strategy enables more effective multi-view feature interaction through the proposed self-attention of view-inside features and cross-attention of view-outside features.

### 3.5. Feature Self-Attention inside the View

In SMIFormer, we utilize the self-attention of view-inside features to promote feature interaction within each view. To achieve this, we first gather sparse features $F_{i}=\left\{f_{1,i},\ldots ,f_{N_{i},i}\right\}$ within each window *i* for a given perspective’s feature map. Each window can be represented as a three-dimensional sparse feature matrix. The spatial shape of these matrices varies depending on the type of view (bird’s-eye view or front/side views). For example, in a bird’s-eye view representation, the spatial shape is $W_{bev}\times H_{bev}\times D$, while it is $W_{fvsv}\times H_{fvsv}\times D$ for front and side views. Here, *D* represents the feature dimensionality per non-empty voxel. In the case of the bird’s-eye view, the features $F_{i}$ within each window form an $N_{i}\times D$ matrix in its three-dimensional sparse matrix representation. It is important to note that these three-dimensional feature matrices are sparse, thus satisfying the condition ($W_{i}\times H_{i})\geq N_{i}$. To further facilitate interaction among all sparse voxels within each view, we calculate feature multi-head self-attention within the view on a per-view basis:

(5) $${\hat{F}}_{i}=MA\left(L_{q}\left(F_{i}+PE\right),L_{k}\left(F_{i}+PE\right),L_{v}\left(F_{i}\right)\right)$$

The linear projection layers, $L_{q}\left(\cdot \right)$, $L_{k}\left(\cdot \right)$, and $L_{v}\left(\cdot \right)$, are employed to generate query, key, and value features for the multi-head self-attention mechanism while maintaining the same semantics. To integrate spatial information into the attention process, we introduce ‘PE’ as a learnable index-based 2D position embedding for each sparse voxel. This embedding is obtained by mapping the indices (i,j) of the sparse voxels through a Multi-Layer Perceptron (MLP).

### 3.6. Feature Cross-Attention outside the View

We aim to propagate sparse voxel information at the view-level across different views after applying self-attention within the view. This allows us to capture more comprehensive three-dimensional spatial information. To achieve this, we employ the attention mechanism using the per-view feature representation. We query the features summarized in the full view of the entire three-dimensional space.

To enhance the fusion of features between each view, we calculate the cross-attention of the external features in the view. Specifically, we group all the divided windows in all views based on their voxel projection in 3D space. When windows from the same voxel block are projected onto the three views, they are grouped together. This grouping enables the interaction of window features from the three views within the group, further enriching the features of each view with information from other views. In the following example, a view is used as the query feature, while other views serve as the key and value features for calculating multi-head cross-attention:

(6) $$\begin{array}{c} {\hat{F}}_{i}^{v_{1}}=MA\left(L_{q}\left(F_{i}^{v_{1}}+{PE}^{v_{1}}\right),L_{k}\left(F_{i}^{v_{2}}+{PE}^{v_{2}}\right),L_{v}\left(F_{i}^{v_{2}}\right)\right)\\ +MA\left(L_{q}\left(F_{i}^{v_{1}}+{PE}^{v_{1}}\right),L_{k}\left(F_{i}^{v_{2}}+{PE}^{v_{3}}\right),L_{v}\left(F_{i}^{v_{3}}\right)\right) \end{array}$$

Each view performs similar calculations to update its own features.

## 4. Experiments

### 4.1. Dataset

In this study, we utilize the View of Delft (VoD) [44] dataset to evaluate the performance of the proposed method. This dataset is designed to validate the performance of radar-based road detection. The dataset also covers multi-modal data from various sensors, including 4D radar points, LiDAR points, and camera images. The VoD dataset provides a corresponding label for each object to be detected, including its category, a 3D bounding box, and a tracking ID. As well as coordinate transformation matrices between different sensors.

In our experiment, we mainly detected three main categories in the dataset, including cars, pedestrians, and cyclists. For the division of the data set, we follow the official split method. Specifically, the VoD dataset comprises 5139 frames for training and 1296 frames for validation. Since the VoD data set does not provide test set labels and automated test set evaluation systems, our tests and analysis are performed on the validation set.

### 4.2. Evaluation Metrics

We evaluated SMIFormer using mean average precision (mAP). The threshold used in the calculation of AP is based on the intersection over union (IoU) of the predicted and ground truth bounding boxes in 3D, which requires an overlap of 0.5, 0.25, and 0.25 for cars (Car), pedestrians (Ped), and cyclists (Cyc), respectively. In the VoD dataset, there are two official evaluation matrices, each specifying different ranges of detection areas: (1) region of the entire annotation area (camera FoV up to 50 m). (2) region of the driving corridor, defined as a rectangle on the ground plane in front of the vehicle as [−4 m < *x* < 4 m, *z* < 25 m] in camera coordinates, which is more relevant to actual driving.

### 4.3. Implementation Details

The model implementation is based on OpenPCDet [45], an open-source framework designed for 3D object detection, and was trained on four NVIDIA RTX A6000 GPUs for 80 epochs; the batch size is set to 4. The point cloud range (PCR) is limited to D(PCR)={(x,y,z)∣ 0 < *x* < 51.2 m, −25.6 m < *y* < 25.6 m, −3 m < *z* < 2 m} in the radar coordinate. The voxel size in the voxelization process for the radar cloud points is set to 0.05 m × 0.05 m × 0.125 m. We implemented Adam as our optimizer, set the initial learning rate to 0.003, weight decay to 0.01, and momentum to 0.9. After we use the proposed method to encode the point cloud data and obtain the BEV feature map, We implement the dense head based on the CenterPoint [2] to output the detected bounding boxes. For data augmentation, because the velocity measured by the radar must remain relative to the angle of the observed object, rotating the bounding boxes and the points inside will change the radial component of the object’s velocity in an unknown way [44]. Therefore, we only use mirror inversion and scaling as augmentation.

### 4.4. Results and Analysis

In this study, we compare SMIFormer with other state-of-the-art models for 3D detection based on point clouds. The experimental results of the VoD [44] validation set are presented in Table 1. To obtain a comprehensive comparison, We deployed some 3D object detection networks designed for LiDAR point clouds on the VoD dataset for evaluation, including anchor-based detectors like PointPillars [29], SECOND [22], and the anchor-free detector CenterPoint [2]. Furthermore, we compare the proposed method with recently proposed 3D object detection methods designed for 4D radar, such as LXL-R [39]. Recently, a multi-modal fusion of camera images and radar point clouds has become mainstream for 3D object detection; thus, we also compared our method with the latest RCFusion [38], which was designed for the feature fusion of 4D radar point clouds and camera images.

Our SMIFormer achieves better results compared with several other detection methods. Compared with CenterPoint, which also uses anchor-free detectors, the proposed method with a three-view fusion encoder has achieved significant improvements, with an increase of 3.35% and 6.07% on mAP across the entire annotation area and driving corridor area. Compared with conventional methods using anchor-based detectors, the proposed method also achieved an improvement of no less than 3.36% and 4.41% in mAP in the two annotated areas, respectively. LXL is a relatively new 3D target detection method based on a 4D radar point cloud, which has achieved optimal results in single-modal detection; our method still outperforms it in terms of mAP, which confirms that our proposed method indeed improves object detection accuracy. At the same time, we found that SMIFormer’s pedestrian detection accuracy is significantly higher than other solutions. This may be because the pedestrian’s bounding boxes are prone to interference and occlusion problems due to their small size. SMIFormer improves pedestrian detection by introducing additional view capabilities to enhance point cloud features associated with small objects.

Despite the fact that SMIFormer only uses a 4D radar point cloud as a single-modal input, compared with the newer radar-camera fusion method RCFusion [38], although the mAP has declined in the entire annotation area, it has achieved significant improvements in the driving corridor area. This may be due to the sparsity of radar points, resulting in some targets’ bounding boxes far away from the radar having very few radar points. The fusion-based detection method uses camera images to supplement the features of these areas, thereby improving the mAP of the entire area. While SIMFormer combines features from different views, it enables the model to have better perception capabilities in relatively dense areas of point clouds, thereby improving detection accuracy in the driving corridor areas.

**Figure 4:**
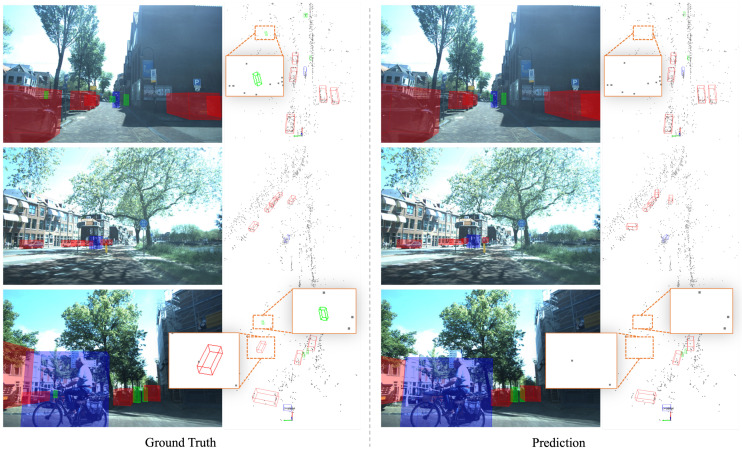
Visualization results on the VoD validation set. Each row contains two sets of data frames of images and radar points (gray points). The left side is the ground truth, the right side is the prediction result, the red box represents the car, the blue box represents the cyclist, and the green box represents the pedestrian. The local region of the point cloud, scaled by the orange wire box, demonstrates that for a few undetected objects, there are few to no points in these ground truth bounding boxes.

We visualize the results of SMIFormer on the vod validation set in Figure 4. We can find that the proposed method can detect the target object in the vast majority of cases, which shows that under the support of the tri-perspective fusion, our method can accurately locate the position of the object. For a small number of undetected objects, we observed that there are few or even no point clouds in these ground truth bounding boxes, which results in the generation of empty voxels and prevents the model from obtaining useful features.

### 4.5. Ablation Study

In this subsection, we performed several experiments on the VoD validation dataset to further demonstrate the effectiveness of our SMIFormer.

#### 4.5.1. Effects of Proposed Components

A comprehensive set of ablation studies is conducted for each of our proposed components, as illustrated in Table 2. VoxelNet [21] is adopted as the baseline, and the Inception sparse convolution block and 2D sparse downsample encoder are consistently employed across all experiments.

Based on the results presented in Table 2, it is evident that the introduction of multi-view plane decoupling into the voxel encoder (MVD, #1 and #2) brings noticeable improvements compared with the baseline. This serves as evidence that incorporating semantic features from different perspective views in the voxel space significantly enhances the model’s ability to represent radar point clouds. Furthermore, the utilization of attention operations further enhances the efficiency of multi-view fusion. Feature self-attention inside the view (SAI, #3) can enhance the global representation of a single view, thereby improving the performance of the network. Additionally, feature cross-attention outside the view (CAO, #4) enhances the network’s ability to represent different perspectives by enabling features from different views to interact in the same semantic space. This integration of features from different perspectives leads to a better overall performance. By combining all of these components (#4), we observe a significant improvement in mAP for both the entire annotation area (from 46.48% to 48.77%) and the driving corridor area (from 68.36% to 71.13%). These results serve as strong evidence for the effectiveness of our proposed method.

#### 4.5.2. Effects of Splitting Window Size

We also investigate the impact of splitting window size on the implementation of feature cross-attention outside the view during the fusion of different views. Window sizes of 2, 4, and 8 are selected, and the model containing all components is evaluated accordingly. The results are presented in Table 3, revealing that the model achieves the highest mAP in both annotation areas when the window size is set to 4. When the window size is reduced to 2, the model’s performance experiences a slight decrease, but it should be noted that a smaller window size leads to increased computational resource requirements during attention calculation per unit of time. Conversely, when the window size is increased to 8, the model encounters a significant drop in performance, particularly in pedestrian detection. This can be attributed to the relatively small bounding boxes of pedestrians. Larger window sizes tend to collect more noise information, thereby affecting the accuracy of detecting small targets.

We conducted an experiment to investigate the impact of different window sizes on inference latency. Specifically, we tested the inference time of SMIFormer using various sizes of splitting windows on a single GPU. The results are presented in Table 4. It is evident that both too-small and too-large window sizes result in increased reasoning delay. When the window size is too small, the number of generated windows increases, leading to a higher frequency of attention calculations and thus longer inference times. Conversely, when the window size is too large, although the total number of windows decreases, the number of query vectors within each window significantly increases, resulting in increased inference latency. Therefore, in our other experiments, we consistently adopted a splitting window size of 4.

## 5. Conclusions

In this study, we proposed SMIFormer, a 3D object detection network based on 4D imaging radar. Compared with the traditional 3D object detection backbone network that converts 3D scene features into bird’s-eye view features, due to the sparsity of radar point clouds, the features of a single perspective will also be very sparse and cannot fully represent the whole 3D scene. SMIFormer additionally decouples the front view and side view features, significantly improves the spatial representation capability of the network, and introduces additional downsampling layers to enable the network to extract more deep semantic information. For the interaction between different views, we propose the MVI module, which extracts richer semantic information from sparse point clouds by integrating self-attention-based intra-view feature interaction and cross-attention-based inter-view feature interaction.

In order to solve the problem of the high computational cost of attention mechanism in high-resolution feature maps, we propose a window calculation strategy that divides the multi-view feature maps into a series of non-overlapping windows. Self-attention is performed on internal sparse features in each window, and cross-attention is performed between multi-view windows only when these windows are projected onto the same voxel. This strategy reduces the amount of calculation while ensuring the effectiveness of feature extraction, thereby improving the accuracy of 3D object detection.

The proposed SMIFormer proposes a new method to enhance feature extraction of extremely sparse point clouds, which may be a potential solution for feature extraction of extremely sparse point clouds. Future works will focus on further improving the network’s feature extraction capabilities from sparse point clouds and utilizing camera images to improve multi-view representation of 4D imaging radar point clouds, which will further improve the accuracy and efficiency of 3D object detection based on 4D radar point clouds.

## Figures and Tables

**Table 1:** Comparison on the evaluation set of VoD dataset. All the approaches are executed with 5-scans radar detection points.

Method	Modality	Entire Annotation Area				Driving Corridor Area			
		Car	Ped	Cyc	mAP	Car	Ped	Cyc	mAP
PointPillars [29]	R	37.24	32.19	66.80	45.41	70.55	43.28	88.13	67.32
SECOND [22]	R	40.40	30.64	62.51	44.52	72.25	41.19	83.39	65.61
CenterPoint [2]	R	32.74	38.00	65.51	45.42	62.01	48.18	84.98	65.06
LXL-R [39]	R	32.75	39.65	68.13	46.84	70.26	47.34	87.93	68.51
RCFusion [38]	R + C	**41.70**	38.95	**68.31**	**49.65**	71.87	47.50	**88.33**	69.23
Ours	R	39.53	**41.88**	64.91	48.77	**77.04**	**53.40**	82.95	**71.13**

**Table 2:** Module’s ablation study on the VoD validation dataset. All the approaches are executed with 5-scans radar detection points on VoD dataset.

#	MVD	SAI	CAO	Entire Annotation Area				Driving Corridor Area			
				Car	Ped	Cyc	mAP	Car	Ped	Cyc	mAP
1				37.82	39.44	62.18	46.48	72.08	49.29	83.71	68.36
2	✓			**41.16**	40.07	61.06	47.43	**78.73**	52.43	74.80	68.65
3	✓	✓		39.32	39.92	63.53	47.59	70.64	**53.79**	**85.57**	70.00
4	✓	✓	✓	39.53	**41.88**	**64.91**	**48.77**	77.04	53.40	82.95	**71.13**

**Table 3:** Splitting window size ablation study on the VoD validation dataset. All the approaches are executed with 5-scans radar detection points on VoD dataset.

Window Size	Entire Annotation Area				Driving Corridor Area			
	Car	Ped	Cyc	mAP	Car	Ped	Cyc	mAP
2	**43.48**	38.59	63.18	48.41	**79.59**	51.10	79.95	70.21
4	39.53	**41.88**	**64.91**	**48.77**	77.04	**53.40**	82.95	**71.13**
8	39.35	35.69	64.49	46.51	77.58	47.98	**86.37**	70.64

**Table 4:** Latency evaluation for different splitting window sizes on a single GPU.

Window Size	Latency
2	68 ms
4	**61 ms**
8	65 ms
32	69 ms
128 (w/o splitting)	90 ms

## Data Availability

Data are contained within the article.
